# Disintegration Test of Health Food Products Containing *Ginkgo Biloba* L. or *Vitex Agnus-Castus* L. in the Japanese Market

**DOI:** 10.3390/medicines2020047

**Published:** 2015-04-23

**Authors:** Naoko Sato-Masumoto, Sayaka Masada, Satoshi Takahashi, Sachiko Terasaki, Yoichi Yokota, Takashi Hakamatsuka, Yukihiro Goda

**Affiliations:** 1 Division of Pharmacognosy, Phytochemistry and Narcotics, National Institute of Health Sciences, 1-18-1 Kamiyoga, Setagaya-ku, Tokyo 158-8501, Japan; E-Mails: nasato@nihs.go.jp (N.S.-M.); masada@nihs.go.jp (S.M.); thakama@nihs.go.jp (T.H.); 2 Toyama Prefectural Institute for Pharmaceutical Research, 17-1, Nakataikoyama, Imizu-shi, Toyama 939-0363, Japan; E-Mails: satoshi.takahashi@pref.toyama.lg.jp (S.T.); sachiko.terasaki@pref.toyama.lg.jp (S.T.); yoichi.yokota@pref.toyama.lg.jp (Y.Y.); 3 Division of Drugs, National Institute of Health Sciences, 1-18-1 Kamiyoga, Setagaya-ku, Tokyo 158-8501, Japan

**Keywords:** OTC crude drug product, health food, ginkgo leaf, chaste tree fruit, disintegration test, Western herb

## Abstract

For many years now, a number of Western herbs have been widely used in health food products in Japan and as pharmaceuticals in Europe. There are few or no mandated criteria concerning the quality of these herbal health food products, thus clarification is warranted. Here, we performed disintegration tests of 26 pharmaceutical and health food products containing the Western herbs ginkgo leaf and chaste tree fruit, in accord with the Japanese Pharmacopoeia. All eight pharmaceutical herbal products found in the European market completely disintegrated within the defined test time, and 11 of the 18 tested herbal products distributed as health foods in Japan disintegrated. Among the incompatible products identified in the Pharmacopoeia test, some products remained intact after incubation in water for 60 min. To ensure the efficacy of Western herbal products sold as health food in Japan, quality control, including disintegration, is therefore recommended, even though these products are not regulated under the Pharmaceutical Affairs Law.

## 1. Introduction

The “Western herbs” used nowadays in Japan are thought to be crude drugs originating in Europe and North America, as described in the Complete German Commission E Monographs’ extensive review of many of these herbs [1]. Some of their extracts, and sometimes powders, are used as the active ingredients of over-the-counter (OTC) products in many countries, including Japan. For example, ginkgo leaf, the leaf of *Ginkgo biloba* L., is widely used to treat dementia and other diseases [2], and, in Europe, its preparation is standardized by the European Pharmacopoeia as a pharmaceutical. Preparations of chaste tree fruit (the fruit of *Vitex agnus-castus* L.) are also distributed as OTC drugs for premenstrual syndrome (PMS) in Europe [3].

These Western herbs are widely used as ingredients in health food products, as capsules or tablets, in Japan, where self-medication is becoming more widespread due to the increasing interest in health; however, most of these products are not permitted to be sold as pharmaceuticals. Our continuing research suggests that many health food products and pharmaceuticals obtained in Japan contain materials of an origin different from the indicated materials [4,5,6,7,8,9,10,11,12,13,14]. This is partly the result of a lack of strict regulation of health food products. Health food products that are sold as capsules or tablets are regulated by Japan’s Food Sanitation Law, the purpose of which is to control the safety of foods. This law does not mandate the origin identification of ingredients of products as long as they are ‘safe’. In contrast, crude drugs used for pharmaceuticals, which are regulated by Japan’s Pharmaceutical Affairs Law, are mandated to identify their origin according to the Pharmacopoeia or specific methods described in the Letter of Approvals. 

In 2007, based on a notification from the Japanese Ministry of Health, Labour and Welfare [15], guidelines were established for the approval of pharmaceuticals, including Western herbs, as an active ingredient. Until recently, few Western herbal products were distributed as pharmaceuticals in the Japanese market.

To ensure the efficacy and safety of Western herbal products, it is important that the correct original plant species is used, that products are manufactured appropriately, and that consistent quality and composition are assured [6]. However, these measures alone are not sufficient for quality control. Disintegration is a quality test required of pharmaceutical tablets and capsules. In the present study, to evaluate the quality of a subset of the health food products available in Japan, we surveyed the disintegration of ginkgo leaf and chaste tree fruit products, the material origins of which were revealed previously [4,5,16]. Analyses were made according to the Japanese Pharmacopeia, and the results were compared with those of pharmaceuticals sold in Europe. To our knowledge, this is the first study to perform a disintegration test of health food products containing herbal extracts obtained in Japan.

## 2. Experimental Section

### 2.1. Materials

Ten commercial OTC ginkgo leaf products distributed as health foods were purchased from a Japanese market, and five OTC ginkgo leaf products were obtained from a European market (Table 1). Five chaste tree fruit products distributed as health foods were purchased from the same Japanese market and three were purchased from a U.S.A. market. Three medicinal chaste tree fruit products distributed in Europe were also included (Table 1). All products used in this study were tested prior to their expiration date.

**Table 1:** The pharmaceutical and health food products containing ginkgo leaf and chaste tree fruit tested in this study.

Material	Product ID	Category	Distributing Country	Product Form
Ginkgo leaf	gl-B	Health food	Japan	Tablet
	gl-C			
	gl-E			Film-coated tablet
	gl-F			
	gl-G			
	gl-J			
	gl-A			Soft capsule
	gl-I			
	gl-D			Hard capsule
	gl-H			
	gl-K	Pharmaceutical	Germany	Film-coated tablet
	gl-L			
	gl-M			
	gl-N			
	gl-O			
Chaste tree fruit	cb-D	Health food	Japan	Film-coated tablet
	cb-E			
	cb-F			
	cb-G			Tablet
	cb-J			Capsule
	cb-H		U.S.A.	Capsule
	cb-I			
	cb-K			
	cb-A	Pharmaceutical	Germany	Film-coated tablet
	cb-B			
	cb-C		Switzerland	

### 2.2. Disintegration Test

The disintegration test was performed using water maintained at 37 °C as the immersion fluid according to the Japanese Pharmacopoeia (JP16) [17]. Tests were carried out using a test apparatus (Model NT-6H, Toyama Sangyo, Osaka, Japan), with a device for raising and lowering the basket of the apparatus into the immersion fluid set to move a distance of 55 mm at a constant frequency (30 cycles/min). The disk of the apparatus was used for the testing of only hard-shell capsules. Six tablets or capsules were tested for each product.

### 2.3. Disintegration Test Criteria

The criteria used with the disintegration test were based on the JP16 [17]. Disintegration tests were carried out for 20 min for capsules, 30 min for uncoated tablets, and 60 min for coated tablets. Six capsules or tablets were placed in the basket as the initial lot. After the basket was lifted from the test fluid at the end of 20-, 30-, or 60-min test, the tablets/capsules were observed. Complete disintegration was defined as the disintegration of all residue except fragments of an insoluble coating or capsule shell, or a soft mass with no palpably firm core. If one or two tablets/capsules failed to disintegrate, the test was repeated with 12 additional tablets/capsules. The test was considered successful if no less than 16 of the total 18 tablets/capsules tested disintegrated [17].

## 3. Results

### 3.1. Disintegration of the Ginkgo Leaf Products

Of the 15 ginkgo leaf products shown in Table 1, five medical products completely disintegrated within the defined test time (Table 2). The remaining 10 products (which are distributed as health foods in Japan) were also tested, but only half disintegrated within the test time (Table 3). In the disintegration tests of the two uncoated tablet products (products IDs: gl-B and gl-C) and those of the two film-coated tablet products (gl-F and gl-G), all six tablets of each product remained intact after incubation in water for 30 and 60 min, respectively. In the tests of the soft capsule product (gl-A), three of the six capsules failed to completely disintegrate within 20 min.

**Table 2:** Disintegration test of ginkgo leaf products distributed as pharmaceuticals.

Product ID	Test Result	Test Time (min)	Time for Disintegration (min)						
			Average	1	2	3	4	5	6
gl-K	○	60	15.3	14.6	14.2	14.8	14.8	17.1	16.3
gl-L	○		13.6	13.7	13.7	12.9	13.7	13.3	14.3
gl-M	○		14.0	13.8	13.8	13.8	13.8	14.8	13.8
gl-N	○		13.8	13.8	12.4	15.0	13.3	13.1	15.2
gl-O	○		18.0	17.7	17.7	18.5	18.5	18.5	17.3

○: All samples disintegrated within the defined test time.

**Table 3:** Disintegration test of ginkgo leaf products distributed as health foods.

Product ID	Test Result	Test Time (min)	Time for Disintegration (min)						
			Average	1	2	3	4	5	6
gl-B	×	30	>60	>60	>60	>60	>60	>60	>60
gl-C	×		>60	>60	>60	>60	>60	>60	>60
gl-E	○	60	14.9	14.4	14.4	14.4	14.5	15.4	16.1
gl-F	×		>60	>60	>60	>60	>60	>60	>60
gl-G	×		>60	>60	>60	>60	>60	>60	>60
gl-J	○		5.6	4.0	5.3	5.3	6.1	6.1	6.6
gl-A	×	20	-	13.0	13.0	13.0	>20	>20	>20
gl-I	○		14.7	10.3	12.3	15.3	15.3	17.0	18.1
gl-D	○		5.9	5.3	5.5	5.5	6.1	6.2	7.0
gl-H	○		4.8	4.6	4.6	4.6	4.6	5.1	5.2

○: All samples disintegrated within the defined test time; ×: Disintegration of samples incompatible with the test criteria; -: not determined.

### 3.2. Disintegration of the Chaste Tree Fruit Products

**Figure 1:**
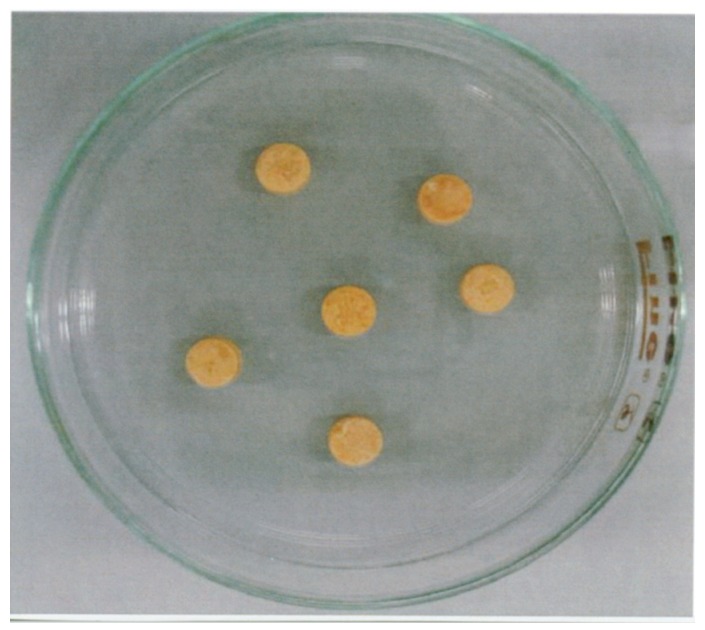
Sample cb-F, 60 min after the disintegration test was started. Product cb-F is a chaste tree fruit product sold as an uncoated tablet.

Disintegration tests of the 11 chaste tree fruit products shown in Table 1 were performed by the same protocol as that used for the gingko leaf products. Three of the chaste tree fruit products disintegrated within the defined test time (Table 4), whereas only six of eight products sold as health foods disintegrated within the allotted time (Table 5). One of the two unbroken products was an uncoated tablet obtained from the Japanese market (product ID: cb-F); it retained its shape after incubation in water for 30 min (Figure 1). The remaining products that did not disintegrate were capsules from the U.S. market (cb-H), three of which did not completely disintegrate within 20 min.

**Table 4:** Disintegration test of chaste tree fruit products distributed as pharmaceuticals.

Product ID	Test Result	Test Time (min)	Time for Disintegration (min)						
			Average	1	2	3	4	5	6
cb-A	○	60	17.6	16.7	16.9	17.1	17.3	17.6	20.1
cb-B	○		15.5	13.3	14.1	15.4	15.5	15.8	19.0
cb-C	○		34.1	26.8	30.9	34.3	35.7	38.3	38.3

○: All samples disintegrated within the defined test time.

**Table 5:** Disintegration test of chaste tree fruit products distributed as health foods.

Product ID	Test Result	Test Time (min)	Time for Disintegration (min)						
			Average	1	2	3	4	5	6
cb-D	○	60	45.4	44.4	45.1	45.1	45.1	45.6	46.8
cb-E	○		30.0	24.4	25.4	27.1	27.7	33.7	41.4
cb-F	×		>60	>60	>60	>60	>60	>60	>60
cb-G	○	30	14.4	10.6	11.7	14.2	15.3	17.1	17.5
cb-J	○	20	7.9	7.5	7.5	8.0	8.0	8.2	8.2
cb-H	×		22.4	14.5	18.0	19.7	22.7	22.7	37.0
cb-I	○		9.6	5.0	5.5	7.5	9.1	14.3	16.0
cb-K	○		5.3	4.8	4.8	5.3	5.3	5.7	5.7

○: All samples disintegrated within the defined test time; ×: Disintegration of samples incompatible with the test criteria.

## 4. Discussion

The disintegration of all pharmaceutical products of ginkgo leaf and chaste tree fruit tested in this study conformed to the criteria of the JP16 disintegration test. In contrast, some ginkgo leaf and chaste tree fruit products distributed as health food products were shown to be incompatible with the JP16 standard; that is, some remained intact after incubation in water for 60 min, raising doubts about their efficacy. One reason for this low disintegration is thought to be as follows. Health food product manufacturers do not want their tablets, which tend to be sold together in bottles, to break during their shipping/storage and, thus, cause consumer dissatisfaction. It is significant that a disintegration test of health food products is not mandated by the Food Sanitation Law, because even if tablets/capsules of health food products do not disintegrate within the digestive tract, their safety but not their efficacy is ensured. On the contrary, disintegration tests of pharmaceuticals are mandated to ensure their efficacy, and this is the reason why all of the pharmaceutical products tested in the present study met the criteria of the test. 

In this study, products gl-A to gl-J correspond to samples A to J in our previous componential analyses of ginkgo leaf products [4,5]. Products gl-A and gl-F were found to include total terpene lactones, one of the main active components in ginkgo leaf extract, also found in pharmaceutical ginkgo leaf products; however, both products were shown to have low disintegrative abilities in the present investigation. Therefore, although the listed components of both products appear to be safe, the desired effects might not be obtained due to their poor disintegration. Our previous studies also revealed that some ginkgo leaf products distributed as health food products contained smaller amounts of ginkgo leaf extract compared to those found in pharmaceutical products, and that they contained potential non-labeled additives, such as quercetin [4,5]. Products, such as gl-J, which we suspected contains artificial additives, are more worrisome since its disintegration was found to conform to the criteria of the JP16 disintegration test, and the use of this product could, therefore, lead to unexpected harmful side effects.

Of the chaste tree products sold as health foods tested herein, some were found to contain more abundant components compared to their levels found in pharmaceuticals, suggesting the possibility of unexpected side effects [16]. In some Western herbal products, the source plant has been found to be different from the defined materials [7,18,19,20]. Our present findings revealed, for the first time, that the disintegration also differs between pharmaceuticals and health food products containing Western herbs. In April 2015, new regulations for health food products sold in Japan will be introduced, allowing some indication of the efficacy of such products if these products have clear evidence of efficacy for health. These health food products are regulated under the Food Labeling Law in addition to the Food Sanitation Law. In this case, disintegration tests are required, but an administrative review is not needed. However, many other health food products that are available to the public are still not required to undergo disintegration testing, and since consumers cannot judge the disintegration of health food products, we propose that the new regulations mandating disintegration testing should be expanded to cover these other health food products that are sold as capsules or tablets, even the products that do not exhibit any efficacy, considering their benefits and risks to consumers. 
